# The Role of Nrf2 and Cytoprotection in Regulating Chemotherapy Resistance of Human Leukemia Cells

**DOI:** 10.3390/cancers3021605

**Published:** 2011-03-29

**Authors:** Stuart A. Rushworth, David J. MacEwan

**Affiliations:** School of Pharmacy, University of East Anglia, Norwich NR4 7TJ, UK

**Keywords:** leukemia, AML, CLL, apoptosis, transcription factor, Nrf2, antioxidant, HO-1, NQO1, glutathione, chemotherapy, NF-κB, acute myeloid leukemia

## Abstract

The Nrf2 anti-oxidant response element (ARE) pathway plays an important role in regulating cellular anti-oxidants. Under normal cellular conditions Nrf2 can be described as an anti-tumor molecule due to its induction of cytoprotective genes which protect cells from electrophile and oxidative damage. However in cancerous cells, Nrf2 takes on a pro-tumoral identity as the same cytoprotective genes can enhance resistance of those cancer cells to chemotherapeutic drugs. Such Nrf2-regulated cytoprotective genes include heme oxygenase-1 (HO-1), which has been shown to protect human leukemia cells from apoptotic signals. Moreover, a relationship between Nrf2 and the nuclear factor-κB (NF-κB) signaling pathway has been recently identified, and is now recognized as an important cross-talk mechanism by which Nrf2 can overcome apoptosis and provide cells with reduced sensitivity towards chemotherapeutic agents. In recent years a number of important research papers have highlighted the role of Nrf2 in providing protection against both current and new chemotherapeutic drugs in blood cancer. This review will provide a synopsis of these research papers with an aim to carefully consider if targeting Nrf2 in combination with current or new chemotherapeutics is a viable strategy in the more effective treatment of blood cancers.

## 1. Introduction

Nrf2, first cloned and characterized by its ability to bind to the NF-E2/AP-1 repeat in the promoter of the β-globin gene [1], is ubiquitously expressed in many organs as a transcriptional activator for cytoprotective and phase II genes by binding to antioxidant responsive element (ARE) [2]. Over the past 10 years the role of Nrf2 has been increasingly studied to show that Nrf2 activation can protect against many human diseases and pathological states such as cancer, neurodegenerative diseases, aging, cardiovascular disease, inflammation, pulmonary fibrosis and acute pulmonary injury [3-7]. Using dietary or synthetic compounds to boost Nrf2-mediated cellular defense responses, Nrf2 has been intensively studied in diseases prevention [8-11]. Many Nrf2 activators have been identified and their efficacy in cancer prevention has been verified both in animal models and in human clinical trials [12,13]. Here, we provide a different perspective, one where the activation of Nrf2 will protect cancer cells from undergoing apoptosis.

Nrf2 functions to rapidly change the sensitivity of a cell's environment to oxidants and electrophiles by stimulating the transcriptional activation of over a hundred cytoprotective and detoxification genes, including the antioxidants ferritin, glutathione-S-reductase (GSR), glutamyl cysteine ligase-modulator (GCLM) and -catalytic (GCLC), phase-I drug oxidation enzyme NAD(P)H:quinone oxidoreductase 1 (NQO1), and cytoprotective enzyme heme oxygenase-1 (HO-1) [2,3,6,14,15]. With respect to HO-1, other transcription factors including nuclear factor-κB (NF-κB) and activator protein-1 (AP-1) are also involved in its expression [16,17]. Under normal physiological conditions, the inhibitor of Nrf2, Keap1 (Kelch ECH Associating Protein 1) mediates ubiquitin-26S proteasomal degradation of Nrf2. Oxidative and electrophilic stresses such as reactive oxygen species (ROS) impair Keap1-mediated proteasomal degradation of Nrf2, causing Nrf2 activation and its subsequent nuclear translocation [18,19]. Nuclear Nrf2 forms a heterodimer complex with Maf proteins which bind the ARE located in the enhancer regions of Nrf2-inducible genes [2]. Therefore, side effects of proteasome inhibition may lead to increased cellular levels of Nrf2 and its activation and up-regulation of its target proteins. However, a regulatory process exists to control nuclear Nrf2 activation in the form of the transcriptional repressor known as Bach1 (BTB and CNC homolog 1), bound to ARE enhancer regions in cells lacking oxidative stress to block Nrf2 binding [20]. Bach1 becomes deactivated and translocates to the cytosol, upon pro-oxidant stimuli.

In order to fully appreciate the impact of Nrf2 signaling in human cancer cells we will give a brief overview of how Nrf2 is activated, followed by an analysis of the nature of the genes up-regulated by Nrf2 activation. We will then talk about how cancer cells have evolved to manipulate Nrf2 signaling in order to protect themselves from undergoing apoptosis in response to chemotherapy. This will be followed by the main focus of the review, the evaluation of the published data regarding Nrf2 in human leukemia and lymphoma. Our overall aim is to inform the reader about the molecular mechanisms underlying Nrf2 deregulation in hematological malignancies and its impact on chemotherapy resistance.

## 2. Nrf2 Activation

Firstly, we will define the nature of Nrf2 activation, the other molecules involved and how they interact with each other to bring about Nrf2 mediated transcription. This will illustrate that understanding the mechanistic processes involved in Nrf2 signaling is essential for determining how best we can modulate the effect of this pathway to suit our requirements in preventing disease processes induced by deregulation of this pathway.

Nrf2 activation depends on Keap1 a cytoskeleton protein capable of binding actin filaments and Nrf2, thus preventing its nuclear translocation and acting as transcriptional repressor during basal conditions. A number of different models of Nrf2 activation have been proposed, including Nrf2 release from Keap1, Nrf2 protein stabilization, Keap1 shuttling and the induction of Nrf2 gene expression: The two models which are generally accepted include:

### 2.1. Nrf2 release from its inhibitor Keap1

Under normal homeostatic conditions, Nrf2 is retained in the cytoplasm through its interaction with Keap1. Upon oxidative stress, Nrf2 is released from Keap1, and translocates from the cytoplasm to the nucleus [18]. This release mechanism has been extensively studied and several mechanisms have been proposed including modification of Cys residues in Keap1, phosphorylation at Ser40 by protein kinase C (PKC), extracellular signal-regulated kinase (ERK), c-Jun N-terminal kinase (JNK) and phosphoinositide-3-kinase (PI3K) [21,22];

### 2.2. Nrf2 protein stabilization

Under unstressed conditions, Nrf2 is constantly degraded via Keap1-mediated ubiquitination that is counter balanced by constitutive Nrf2 translocation. Specifically, in addition to binding Nrf2 through its Kelch-repeat domain, Keap1 also binds Cullin-3 (Cul3) [23,24]. In turn, Cul3 binds its partner Rbx1 to form a core E3 ubiquitin ligase complex. This complex targets Nrf2 for degradation. Under oxidative conditions, Keap1-mediated ubiquitination is impeded, while Nrf2 nuclear translocation levels are elevated, thus resulting in binding to the ARE in combination with other mediators of transcription.

After migration to the nucleus, Nrf2 undergoes heterodimeric combinations with other transcription factors, such as small Maf protein and binds to the 5′-upstream *cis*-acting regulatory sequence, termed ARE or electrophile response elements. However, the role of Nrf2-Maf heterodimers in ARE-mediated gene regulation is controversial and requires further investigation [2,25]. Over the past decade evidence has been accumulating that suggests another control mechanism of Nrf2 activation exists. This is in the form of another transcription factor Bach1 which is known to bind to the Nrf2 site as a heterodimer with Maf [20,26]. Upon induction, Bach1 is replaced by Nrf2, resulting in activation and suggesting competition between Bach1 and Nrf2 for the same DNA binding site in different cellular states [27,28].

This section shows the many studies which have come together to dissect and understand the mechanism involved in regulating Nrf2 activation. In the next section we will inform the reader about the genes that are regulated by Nrf2 activation together with what this means for the cellular environment.

## 3. Nrf2 Target Genes

The effects and activities of some of the major Nrf2-target genes will be discussed below. It is difficult to identify which particular genes induced by Nrf2 are most important for its cytoprotective role, but there is no doubt that the coordinated induction of Nrf2-target genes has a dramatic effect on cellular homeostasis.

### Heme oxygenase-1 (HO-1)

HO-1 is arguably the most well known of all Nrf2-regulated genes. It catalyzes the rate-limiting step in the catabolism of the pro-oxidant heme to carbon monoxide, biliverdin, and free iron [29]. HO-1 can have both antioxidative and anti-inflammatory effects, as biliverdin can be reduced to the antioxidant bilirubin, by biliverdin reductase, and carbon monoxide can have anti-inflammatory and apoptotic effects [30,31]. HO-1 mRNA and protein expression are commonly up-regulated following oxidative stress and cellular injury [32], and Nrf2 has been shown to directly regulate HO-1 promoter activity [14,33]. However, other mechanisms of transcriptional regulation are known to exist for HO-1 [34]. For example, Bach1 is a heme-sensing protein that binds to and inhibits Maf proteins, the crucial heterodimer partner for Nrf2 to bind to an ARE [26,35]. In addition, we and others have shown that NF-κB and AP-1 can also regulate HO-1 transcription. Moreover, Kirino and colleagues have shown that the pro-inflammatory cytokine tumor necrosis factor-α (TNF) can induce down regulation of HO-1 in human monocytes by promoting the degradation of HO-1 mRNA [36]. Therefore, HO-1 is regulated by multiple mechanisms in addition to Nrf2, suggesting that other Nrf2-target genes could be quantified as markers for Nrf2 activation.

### NAD(P)H:quinone oxidoreductase 1 (NQO1)

NQO1, also known as DT-diaphorase, is a flavoprotein that is known to catalyze two electron reduction of a broad range of substrates [37]. It is also regarded as a prototypical Nrf2-target gene. NQO1 is critical for cytoprotection against many highly reactive and potentially damaging quinines. This is highlighted in Nrf2 null mice which have reduced constitutive expression and activity of NQO1 in liver, forestomach and small intestine [38]. Furthermore, a non-synonymous mutation in the NQO1 gene, leading to absence of enzyme activity, has been associated with an increased risk of myeloid leukemias and other types of blood dyscrasia in workers exposed to benzene [39].

### Glutamate-cysteine ligase (GCL)

GCL is an enzyme composed of two subunits: a modifier subunit (GCLM) and a catalytic subunit (GCLC), both of which contain ARE sequences in their promoters [11,12]. GCL performs the rate-limiting and ATP-dependent step in glutathione (GSH) synthesis by catalyzing the formation of γ-glutamyl cysteine from glutamine and cysteine. GSH maintains intracellular redox balance and protects against oxidative stress. Additionally, GSH can detoxify chemicals through direct binding or enzymatic conjugation by glutathione-S-transferase (GST) enzymes [40,41]. GSH also plays an important role in free radical scavenging.

### Glutathione-S-transferases (GST)

*GST* catalyze the nucleophilic attack by reduced GSH on nonpolar compounds that contain an electrophilic carbon, nitrogen, or sulfur atom, often resulting in detoxification. GST mRNA and protein expression are decreased in Nrf2-null mice, and Nrf2 is required for GST induction [42]. Moreover, the mRNA expression of GST is markedly increased in Keap1-null mice [43].

### Multidrug resistance-associated proteins (MRP)

MRP are ATP-dependent efflux transporters that export a wide-range of substrates, but especially glutathione, glucuronide, and sulfate conjugates. Four (MRP2, 3, 4, and 6) of the eight MRP expressed in mice are expressed in liver. Furthermore, Mrp2, 3, 4, and 6 in liver are induced by several Nrf2 activators, namely BHA, oltipraz, and ethoxyquin [44,45] However, Keap1-knockdown mice which have a whole body partial knockdown of Keap1 have only a modest induction of MRP2 mRNA expression in liver and no increase in hepatic MRP2 protein expression [43]. This observation, coupled with another study that demonstrated the capability of other transcription factors, such as NF-κB, contribute to MRP regulation, suggests that other regulatory mechanisms might control MRP induction [46,47].

This is by no means anywhere near a full list of the genes that are regulated by Nrf2, indeed there are over a hundred genes regulated by this process and we cannot possibly include all in this review. However, what this section has shown is the nature of the genes that are regulated by this signaling mechanism, and how cancer cells might be able to manipulate the function of these genes in order to provide their anti-apoptotic advantage during chemotherapy treatment.

## 4. Nrf2 and Cancer

In this section, we want to determine how Nrf2 signaling is manipulated so that it and its associated genes can provide protection to cancer cells. To date, numerous mutations have been found of both Keap1 and Nrf2 in human cancers leading to constitutive expression of pro-survival genes. The prognosis of patients with either Nrf2 or Keap1 mutations, which lead to increased expression of cytoprotective genes is much lower than patients with no mutation [48,49]. Moreover, it is only now becoming clear that the number of mutations in this pathway within different cancers is very high and new findings are being reported with increasing occurrence.

Examples of Keap1 mutations include findings by Padmanabhan *et al.* who identified mutations of Keap1 in tissues or cell lines derived, from lung cancer patients [50]. Because of the reduced affinity to Nrf2, these mutant Keap1 proteins could not repress Nrf2 activity and, consequently, Nrf2 was constitutively activated in these cancer cells. Similarly, multiple somatic mutations have now been identified in lung cancer cell lines and non-small-cell lung cancer samples [51,52]. Decreased Keap1 activity in these cancer cells induced greater nuclear accumulation of Nrf2 and constitutive over expression of ARE-containing genes including MRP, NQO1 and GCL. All these proteins have been shown to facilitate resistance of tumor cells to chemotherapy. Keap1 mutations have also been found in breast and gall bladder cancers [53].

Nrf2 mutations have also been identified in various human cancers. Shibata *et al.* identified Nrf2 somatic mutations in patients with lung cancers, head and neck tumors, adenocarcinomas and large cell neuroendocrine carcinoma [49]. Most of these mutations led to impairment of their recognition site for Keap1, which led to the continuous activation of Nrf2. Moreover, it has been suggested that patients with lung tumors containing mutant Keap1 or Nrf2 show a poorer prognosis than patients with non-mutant tumors [49,53]. Therefore, in tumors, inhibition of Nrf2 can be expected to repress tumor cell proliferation and enhance apoptosis. Several reports have demonstrated that administration of Nrf2 targeted siRNA into cancer cells could decrease the growth rate of cells [54,55]. Further studies are needed to unravel the role of Nrf2 in cell proliferation, growth and apoptosis, which can account for a positive correlation of Nrf2 over expression and tumor growth.

Another emerging role of Nrf2 is on process of regulating proteins involved in the process known as autophagy. Autophagy is a highly conserved process involving the degradation of long-lived proteins by lysosomal hydrolysis. The protein p62 is a selective substrate for autophagy, regulating the formation of protein aggregates, and excess p62 has been shown to be involved in human cancer. Moreover, with disturbances in the process of autophagy, p62 can accumulate and activate Nrf2 by competing with Nrf2 for its binding to Keap1 [56,57]. This is a major new insight into how Nrf2 may become activated in malfunctioning cancer cells and the subsequent response to cancer chemotherapy.

In this section we have described how mutations in both Keap1 and Nrf2 provide malignant cells with the capacity to protect themselves from harmful or apoptotic effects, thus representing a key survival factor in different types of cancer.

## 5. Nrf2 in Leukemia

The main focus of the review is to evaluate the published data about Nrf2 in human leukemia. Below we will provide an introduction to blood cancer, followed by how Nrf2 has become a very useful target in combination with conventional chemotherapy for certain leukemias. Moreover, the lack to date of published data on Nrf2 in certain blood cancers suggests that either Nrf2 has no impact on these cancers or that the impact has yet to be properly discovered.

Blood cancers such as leukemia, Hodgkin's lymphoma, NHL, myeloma and MDS are cancers that originate in the bone marrow or lymphatic tissues. They are considered to be related cancers because they involve the uncontrolled growth of cells with similar functions and origins. The diseases result from an acquired genetic injury to the DNA of a single cell, which becomes malignant and multiplies continuously. The accumulation of malignant cells interferes with the body's production of healthy blood cells. Every four minutes one person is diagnosed with a blood cancer with an estimated 54,020 people dying from this disease in the United States in 2010. These blood cancers will account for nearly 9.5 percent of the deaths from cancer in 2010. An estimated 957,902 people in the United States are currently living with, or are in remission from leukemia, Hodgkin lymphoma, NHL or myeloma. The risk for recurrence often depends on the diagnosis, stage, characteristics of disease, the treatments used, when the treatments were given and underlying risk factors independent of the cancer or its treatment.

Not much is known about the role of Nrf2 in blood cancers especially in Hodgkin lymphoma, non-Hodgkin lymphoma, myeloma and myelodysplastic syndromes. However, studies are starting to emerge regarding the effectiveness of Nrf2 regulating survival of cancer cells in leukemias such as chronic myeloid leukemia (CML), acute myeloid leukemia (AML) and chronic lymphocytic leukemia (CLL) [54,55,58-62]. Below we will discuss these emerging reports with respect to each of these diseases as well as trying to put in perspective the incidence of Nrf2 in promoting survival or preventing apoptosis of leukemia cells.

### Chronic myeloid leukemia (CML)

CML is a hematopoietic stem cell disease defined by the presence of the Philadelphia chromosome, which is generated by the reciprocal translocation t(9;22) [63]. The resulting fusion of the c-Abl- and BCR genes generates the BCR/Abl oncogene, which encodes a 210 kDa oncoprotein, BCR/Abl, exhibiting constitutive Abelson tyrosine kinase (TK) activity [64]. An important mechanism in the pathogenesis of CML appears to be enhanced survival and consequent accumulation of leukemic cells [65]. Correspondingly, BCR/Abl has been described to promote expression of a number of antiapoptotic molecules in leukemic cells [66].

During the past few years, imatinib (Gleevec) has become frontline therapy against CML and has been shown to be superior in producing complete cytogenetic and molecular responses compared with other drugs [67]. However, resistance against imatinib can occur during therapy with imatinib, particularly in accelerated phase (AP) and blast phase (BP) CML, as well as in acute lymphoblastic leukemia (ALL), and can represent a serious clinical problem [68,69].

With regards to Nrf2 in CML, Bonovolias *et al.* have recently shown that Nrf2-regulated genes can counteract imatinib-induced apoptosis [60], Moreover a number of studies have revealed that HO-1 provides resistance to imatinib and other CML targeted drugs and that silencing HO-1 removes the resistant nature of these cells [70,71]. Moreover, the Nrf2-regulated genes NQO1 and glutathione have both been linked to providing protection against apoptosis in human CML [72,73]. These data suggest that Nrf2 and its associated cytoprotective genes can protect CML from chemotherapy-induced apoptosis.

### Acute myeloid leukemia (AML)

AML is one the most common malignant hematopoietic disorders in adults. It comprises a heterogenous group of clonal disorders of hematopoietic progenitors, showing genetic instability. Although conventional chemotherapy regimens often ablate actively cycling leukemic blast cells, the primitive leukemic stem cell population is likely to be drug-resistant. Moreover, in AML currently available drugs do not effectively distinguish between malignant stem cells and normal hematopoietic stem cells. Leukemic stem cells, which are quiescent or slowly cycling and therefore less sensitive to chemotherapy, are responsible for disease relapse and represent the target for future innovative therapies [74-76]. Furthermore, with 75% of patients diagnosed after the age of 60 and with current intensive chemotherapeutic strategies generally limited to a minority of younger, fitter patients there is a significant unmet need for better tolerated, more widely applicable and targeted anti-AML chemotherapy [77]. Our goal is to exploit unique properties of leukemic cells to induce apoptosis in the leukemic stem cell population, while sparing normal stem cells. One very important difference between normal and leukemic stem cell populations is constitutive NF-κB activation, which has therefore become a target for anticancer drug development [77,78].

NF-κB is an inducible transcription factor and central coordinator of immune responses. In addition to these functions, NF-κB signaling plays a critical role in cancer development and progression NF-κB pathway has been extensively studied in AML and been found to be constitutively activated in AML blast cells derived from patients [78,79]. Further, increased NF-κB activity has been strongly correlated with AML blast cell count [80]. Since then, several studies have explored targeting NF-κB activity in AML. For example, a study using high concentrations of an IKK2 (inhibitor of κB kinase-2) inhibitor showed induced apoptosis in three out of 15 AML samples [79]. The NF-κB pathway can also be inhibited indirectly by agents that target the proteasome, such as bortezomib and PR-171, but studies in AML show that bortezomib and PR-171 can induce only limited cell death [78]. The use of such NF-κB inhibitors in these studies has been disappointing, implying a limited role for targeting NF-κB transcription factor in AML. The other type of inhibitor of this pathway is targeted to blocking IκB kinase phosphorylation. This too has had limited success, which is highlighted in our studies using BAY-11-7082 (inhibitor of IκB phosphorylation), where AML-derived cell lines HL60, THP-1 and U937 revealed that inhibiting NF-κB had little effect on inducing apoptosis in these AML cells lines and primary samples [17,55]. Clearly other pathways must be targeted to discover more effective AML therapies.

To date, little is known regarding the role of Nrf2 in AML. Our own investigations have shown that in AML-derived cells, but not primary cells, HO-1 is up-regulated in response to TNF stimulation in conjunction with NF-κB inhibition. Furthermore, this induction of HO-1 protects AML cells from cell death signals [55]. The signaling machinery involved in regulating this response is Nrf2. HO-1 and Nrf2 may protect against the detrimental effects of inflammation and oxidative stress but may also help protect cancerous AML cells from TNF-mediated cell death, resulting in clinically devastating levels of apoptosis resistance. Moreover, our work and a study by a Japanese group have revealed that Nrf2 is constitutively active in some AML samples but not all suggesting that mutations may well be found in either Keap1 and/or Nrf2 in AML in the near future [61,81]. The study by Miyazaki *et al.* showed that constitutive Nrf2 induces HO-1 which provides protection for AML cells from chemotherapy-induced apoptosis [61] which will be discussed in more detail in the next section.

### Chronic lymphocytic leukemia (CLL)

CLL is the most common form of leukemia in the Western world. Despite advances in our understanding of the biology of CLL, its current management includes monitoring without treatment of asymptomatic patients until disease progresses by clinical criteria. Improving the care of slowly progressing CLL will require the development of compounds with minimal or no toxicity. Recent studies have shown that drugs which induce reactive oxygen species are not as potent at inducing cytotoxicity of CLL, and since Nrf2 signaling pathway regulates the oxidative stress response [82], it would be fair to implicate Nrf2 in the protection of cells against drugs which induce reactive oxygen species. A study by Wu *et al.* showed that untreated CLL had elevated Nrf2 levels when compared with normal lymphocytes [58]. Moreover, 27 known electrophilic and antioxidant compounds with drug-like properties were tested in for cytotoxic effects on CLL and specifically on targeting Nrf2 signaling. The selected compounds were from five distinct structural classes; alpha-beta unsaturated carbonyls, isothiocyanates, sulfhydryl reactive metals, flavones, and polyphenols. There results showed that compounds containing alpha-beta unsaturated carbonyls, sulfhydryl reactive metals, and isothiocyanates are strong activators of Nrf2 in a reporter assay system and in primary human CLL based on increased expression of the Nrf2 target HO-1. Alpha-beta unsaturated carbonyl-containing compounds were selectively cytotoxic to CLL, and loss of the alpha-beta unsaturation abrogated Nrf2 activity and CLL toxicity. The alpha-beta unsaturated carbonyl containing compounds ethacrynic acid and parthenolide activated Nrf2 in normal peripheral blood mononuclear cells, but had a less potent effect in CLL cells. Furthermore, ethacrynic acid bound directly to the Nrf2-negative regulator Keap1 in CLL cells. These experiments document the presence of Nrf2 signaling in human CLL and suggest that altered Nrf2 responses may contribute to the observed selective cytotoxicity of electrophilic compounds in this disease.

In this section we have evaluated the existing data regarding Nrf2 and its associated genes protecting leukemia and lymphomas from undergoing apoptosis in response to endogenous signals as well as to chemotherapeutic drugs. We have hopefully demonstrated that new drugs or ways to manipulate the role of Nrf2 in these cancers are required.

## 6. Nrf2 and Its Role in Modulating Chemo Resistance in Human Leukemia

The dramatic improvement in managing blood cancers is mainly the result of chemotherapy, usually in combinations of two or more drugs. More than 50 different drugs are now used to treat people with blood cancers and a number of potential new therapies are in clinical trials. During the past decade, several important new drugs, and new uses for established drugs, have greatly improved blood cancer cure and remission rates for many people. Today, there are several new classes of drugs with different mechanisms of action. Some of the newer classes of drugs are: BCR-ABL tyrosine kinase (TK) inhibitors, such as imatinib mesylate, dasatinib and nilotinib; histone deacetylase (HDAC) inhibitors such as vorinostat; hypomethylating or demethylating agents such as azacitidine and decitabine; immunomodulators such as lenalidomide and thalidomide; monoclonal antibodies such as CD20-targetted rituximab; and proteasome inhibitors such as bortezomib [71,83-85]

Evidence is now accumulating for the frequent mutation of either Keap1 or Nrf2 in human cancers. Such mutations lead to constitutive expression of cytoprotective genes and detoxification genes, which provide growth advantages and resistance to apoptosis, thus providing chemo-resistance during therapy. The resistance to chemotherapy by the constitutive activation of Nrf2 can be split into three mechanisms of action including:

1. *Over expression of genes involved in drug efflux pumps*. An important mechanism involved in multidrug resistance is the increased activity of efflux pumps, such as those of the MRP. The role of Nrf2 in regulating MRP which provides resistance of leukemia cells to new or conventional systemic anti cancer therapies (SACT) is not yet understood. However, it is hoped that future research will soon shed light on this process.
2. *Over expression of detoxification genes*. Potentially of equal importance in chemo-resistance in leukemia is detoxification by phase II conjugating enzymes, for example GST and UDP-glucuronosyltransferases and NQO1. Inhibitors of GST and NQO1 have both been shown to enhance the cytotoxicity of established chemotherapies in human AML cells [86,87]. Moreover, a synergistic interaction between MRPs and phase II enzymes, in conferring multidrug resistance has been shown for multiple anticancer drugs. In addition, there is substantial evidence of a coordinate regulation of the expression of phase II enzymes and MRPs, most likely mediated by Nrf2 [87].
3. *Over expression of antioxidant genes*. The main antioxidant gene regulated by Nrf2 is HO-1. HO-1 is critical in two important physiologic processes: recycling of iron molecules for erythropoiesis and maintaining homeostasis under stressful conditions [29]. The role of HO-1 in cancer biology is far from understood. We have shown that AML cells have low HO-1 expression when compared with non-malignant control cells [17]. Interestingly, expression of HO-1 is usually increased in solid tumors compared with surrounding healthy tissues [88]. In addition, primary CML cells express HO-1 in a constitutive manner, and BCR/Abl fusion protein was found to upregulate HO-1 production in CML cells [71]. Further work showed that targeting HO-1 activity concurrently with imatinib treatment induced growth arrest in CML patient cells and imatinib-resistant CML cells were killed by blocking HO-1 activity. In our study, we showed that AML samples become susceptible to apoptotic processes in the presence of combined NF-κB and HO-1 inhibition [17].

Nrf2 signaling is now becoming an established pathway which contributes to the development of acquired resistance to new and old systemic anti-cancer therapies in human leukemia [89,90]. In our own studies we have seen that primary AML cells have constitutive activation of Nrf2 which provides protection against new treatments such as proteasomal inhibitors, as well to established agents, for example daunorubicin and cytarabine [79 and unpublished observations]. Independently of our own studies, others have shown that both AML and CLL have constitutive Nrf2 activation and these contribute to any drug-resistant nature of these diseases [58,61].

## 7. Conclusions

It has been shown in this review that the transcription factor Nrf2 has an important role in cancer cell survival through its capacity to regulate the expression of many genes that modulate apoptosis, cell survival and proliferation, as well as inflammation, tumor metastasis and angiogenesis. Several indications of constitutive Nrf2 activation in lymphoid and myeloid malignancies have been reported to date, suggesting a role for this transcription factor in cancer progression. Considering Nrf2 activation as a possible target in CLL and AML may constitute an additional therapeutic strategy for these diseases as well as others which have yet to be considered. Nrf2 inhibitory molecules may be clinically useful, either as single agents or in combination with classical chemotherapeutic agents—such clinical studies are warranted, if not overdue.
